# An Immunological Perspective of Circulating Tumor Cells as Diagnostic Biomarkers and Therapeutic Targets

**DOI:** 10.3390/life12020323

**Published:** 2022-02-21

**Authors:** Eunice Dotse, King H. Lim, Meijun Wang, Kevin Julio Wijanarko, Kwan T. Chow

**Affiliations:** 1 Department of Biomedical Sciences, City University of Hong Kong, Hong Kong 999077, China; edotse2-c@my.cityu.edu.hk (E.D.); kinglim4-c@my.cityu.edu.hk (K.H.L.); meijwang-c@my.cityu.edu.hk (M.W.); 2 Department of Paediatrics, University of Melbourne, Parkville, VIC 3010, Australia; kwijanarko@student.unimelb.edu.au; 3 Murdoch Children’s Research Institute, Royal Children’s Hospital, Parkville, VIC 3052, Australia

**Keywords:** circulating tumor cells (CTCs), metastasis cascade, immune cells, immune system, immunotherapy

## Abstract

Immune modulation is a hallmark of cancer. Cancer–immune interaction shapes the course of disease progression at every step of tumorigenesis, including metastasis, of which circulating tumor cells (CTCs) are regarded as an indicator. These CTCs are a heterogeneous population of tumor cells that have disseminated from the tumor into circulation. They have been increasingly studied in recent years due to their importance in diagnosis, prognosis, and monitoring of treatment response. Ample evidence demonstrates that CTCs interact with immune cells in circulation, where they must evade immune surveillance or modulate immune response. The interaction between CTCs and the immune system is emerging as a critical point by which CTCs facilitate metastatic progression. Understanding the complex crosstalk between the two may provide a basis for devising new diagnostic and treatment strategies. In this review, we will discuss the current understanding of CTCs and the complex immune-CTC interactions. We also present novel options in clinical interventions, targeting the immune-CTC interfaces, and provide some suggestions on future research directions.

## 1. Introduction

Circulating tumor cells (CTCs) are a heterogeneous population of tumor cells that have shed from a tumor into the lymphatics and vasculature, ultimately disseminating into blood circulation [1]. They have been increasingly studied in recent years due to their clinical relevance. As a component of liquid biopsies, CTCs are important for diagnosis, prognosis, and monitoring treatment responses [2]. Currently, CTCs are the only cell type with commercially available assays approved for use in cancer-related treatment decisions [3]. Beyond their clinical significance, CTCs offer a platform for transcriptomics, genomics, and proteomic studies to provide valuable information on cancer resistance mechanisms [4]. Studies on CTCs have broadened our understanding on intra-tumor heterogeneity, invasion, metastasis tropism, and immune evasion mechanisms [5].

Interplay between CTCs and the immune system has steadily attracted research interest for its emerging utility in predicting treatment outcomes [6]. 

Notably, CTCs express immune checkpoint regulators and markers that can aid them in evading immune surveillance and alter immune response to improve odds for CTCs survival and metastasis [7,8]. In light of this, further exploration in CTC–immune crosstalk will allow for a better grasp in understanding how CTCs respond towards treatments such as immunotherapy. Ultimately, this will be beneficial for devising novel strategies and interventions to monitor and target immune response against CTCs, contributing to the curb against disease recurrence and metastatic spread.

No doubt, the immune system plays a vital role in tumorigenesis, and CTCs have been known to interact with immune cells in circulation, where they must evade or suppress immune surveillance. This interaction modulates the capacity of CTCs to facilitate metastatic progression. Therefore, understanding the complex immune–CTC crosstalk may provide a basis for designing new treatment strategies.

In this review, we discuss the clinical relevance of CTCs as targets and biomarkers in cancer therapy. We review the crosstalk between CTCs and different immune cell types and highlight the various mechanisms employed by CTC–immune cell clusters to facilitate the metastasis process. We further accentuate the technological advances in CTC isolation and detection strategies, discussing challenges associated with current methods and emphasizing recent efforts for CTC analysis to understand their complexity and heterogeneity. This review provides a forward outlook for scientists and clinicians to address key aspects of CTC–immune interactions that can be harnessed for disease management and treatment.

## 2. Circulating Tumor Cells

Circulating tumor cells were first observed in a metastatic cancer patient by Thomas Ashworth in the mid-nineteenth century [9,10]. They are round in shape and range from 12 to 25 μm in size [11]. Circulating tumor cells express epithelial cell adhesion molecule (EpCAM) and/or cytokeratins (CK), stem cell-like markers, and lack panleukocyte marker CD45 [12,13]. They are fragile and rare [14] (5–50 CTCs in 7.5 mL blood) [15], with a half-life of less than 2.5 h in circulation [16,17]. Yet it has been shown that only few CTCs are required to survive the harsh conditions in the bloodstream and extravasate to seed secondary tumors [18,19,20]. While most CTCs exist in single form, some move as multicellular clusters and can be homotypic or heterotypic [21,22]. Circulating tumor cell clusters in circulation are known to offer survival advantages, with greater metastatic propensity in both human and animal models [21,23].

### 2.1. Single CTCs and CTC Clusters

Single CTCs and clusters are found to have different properties. Single CTCs are derived from a single migratory cell progeny, whereas CTC clusters, found in patients with solid tumors, are of oligoclonal origin [21]. These CTC clusters express more mesenchymal than epithelial markers, as compared to single CTCs [19]. In circulation, CTC clusters make up only 2–4% of the total CTC population but are 50 times more capable of forming metastatic lesions than single CTCs [21,24]. Circulating tumor cell clusters exhibit embolus/thrombus-like behavior in circulation, with much slower flow rate than single CTCs [24]. Morphologically, CTC clusters are irregular [25], and the size of individual cells within the clusters is smaller than single CTCs [26]. The junctional cell–cell protein plakoglobin is highly expressed in CTC clusters [21]. Studies have indicated that CTC clusters are transcriptionally activated, and hypomethylation of transcription factors that regulate genes associated with proliferation and stemness has been reported [27].

### 2.2. CTC and Immune Cell Clusters

Association of CTCs with immune cells has been shown to increase their metastatic potential by enhancing viability and fostering proliferation [22,28]. Neutrophils are among immune cell populations that form clusters with CTCs, through vascular cell adhesion protein 1 (VCAM-1)-dependent intercellular junctions. The CTC-neutrophil clusters express genes that drive cell-cycle progression [22]. Interaction between CTCs and neutrophils occurs within the primary tumor microenvironment before detachment into the bloodstream [22,29]. Cluster formation between CTCs and polymorphonuclear myeloid-derived suppressor cells (PMN-MDSC) has also been reported. Of note, MDSC infiltrates within the tumor microenvironment, promotes tumor growth and inhibits cytotoxic T-cell activity. In circulation, PMN-MDSCs interact directly with CTC to augment dissemination and metastasis by utilizing Notch–Nodal signaling pathway [29]. Circulating tumor cells have also been found to interact with platelets minutes before their dissemination [30]. Circulating tumor cells express thrombin to induce platelet aggregation (cloaking), which promotes arrest in the vasculature and facilitates extravasation from vessel walls [31]. Additionally, cloaking offers CTCs protection from fluid shear stress during initial entry into circulation [32,33]. Circulating tumor cells are also known to interact with other immune cells, such as monocytes [34,35], fibroblast [36] and macrophages [11,37,38].

### 2.3. Tumor-Derived Circulating Hybrid Cells

Tumor-derived hybrid cells (CHCs) formed by cell fusion have been found in the tumor microenvironment and circulation [39]. Cell fusion is the combination of homotypic or heterotypic cells into a single cell, with shared cytoplasmic and nuclear contents [40]. Tumor-derived hybrid cells co-express pan-leukocyte antigen CD45 and tumor markers, indicating that leukocytes are a fusion partner with tumor cells [39]. Among leukocyte populations, macrophages are the major fusogenic cell type in both mouse and human studies [41]. Accordingly, most CHCs express macrophage markers. Studies have shown an upregulation of genes in pathways associated with metastasis, including RUNX1, leukocyte cell adhesion molecule, and FLT4, in these hybrids [42]. Tumor-derived hybrid cells are more common in circulation than single CTCs and retain all functional attributes (genotypic and phenotypic) of the parent cells. They are also known to be highly migratory and invasive, and enhance chemotherapy resistance [39,43].

### 2.4. Cancer-Associated Macrophage-like Cells

While not considered CTCs, cancer-associated macrophage-like cells (CAMLs) are a circulating stromal cell subtype found in the peripheral blood of patients with different solid cancers [44]. These cells are polynucleated and highly differentiated with vacuoles containing tumor material [11]. They are large with varying morphology and range from 25 to 300 μm in size^40^. The CAMLs are postulated to originate from tumor-associated macrophages (TAMs) that have undergone macrophage–macrophage fusion and internalized dying tumor cells before reentering circulation [11]. Cancer-associated macrophage-like cells are known to express a typical macrophage antigen (CD14) and are distinguished from CTCs by their CD45 positivity [39,45]. Unlike CHCs, CAMLs retain their immune cell identity [39]. Like CTCs, the number of CAMLs increase with disease progression [46].

## 3. Metastatic Cascade: Crosstalk between CTCs and the Immune System in Non-Tumor Microenvironment

**Figure 1:**
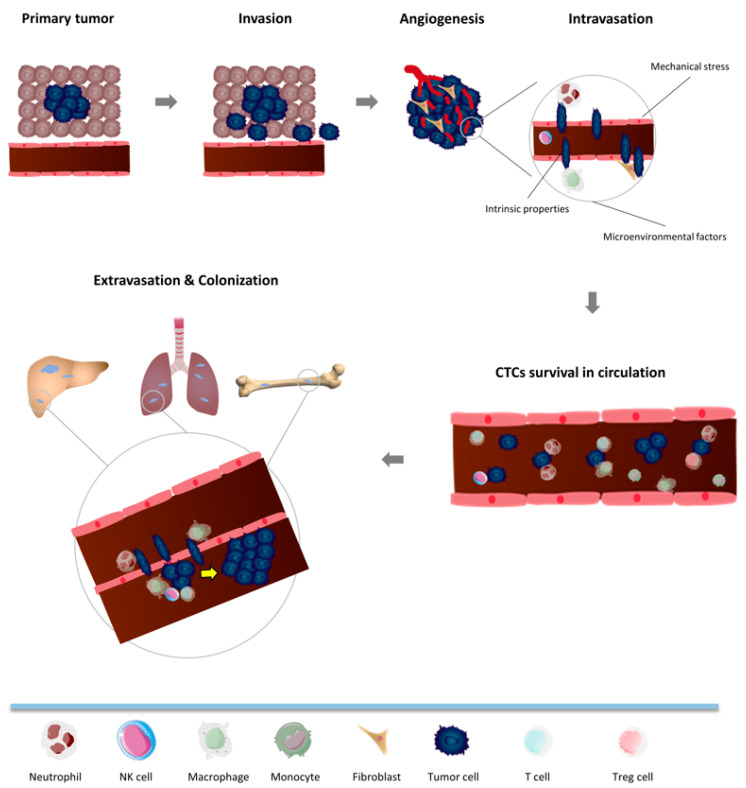
Metastasis Cascade: Interaction between CTCs and Immune Cells.

Circulating tumor cells are a key player in cancer metastasis, a multi-step and complex process that involves (1) local invasion of primary tumor cells into adjacent tissue; (2) intravasation (trans-endothelial migration into nearby blood vessels); (3) circulation (transient travel and survival in the circulatory system as CTCs); (4) extravasation (CTC migration out of the circulatory system followed by seeding in distant organs); (5) colonization (CTC proliferation and growth at seeding sites, eventually becoming clinically detectable metastatic tumors [47,48,49]) (Figure 1). To successfully metastasize, CTCs must evade immune surveillance at every step once they leave the immunosuppressive tumor microenvironment. On the other hand, immune cells can promote or inhibit tumorigenesis, depending on the cell type and context [50]. We hereby focus on the association of immune factors to the metastasis process and highlight the current understanding and unresolved issues in the field.

Metastasis consists of a series of steps including (1) local invasion of primary tumor cells into adjacent tissue; (2) intravasation (trans-endothelial migration into nearby blood vessels); (3) CTCs (transient traveling and survival in the circulatory system); (4) extravasation (CTCs travel out of the blood vessel and seed in bone marrow or other distant organs); (5) colonization (CTCs proliferate and grow at the distant sites, eventually becoming clinically detectable metastatic tumors). CTCs directly interact with immune cells in the circulation and finally escape from immune surveillance.

### 3.1. Intravasation

Intravasation is the process by which cancer cells shed from primary or metastatic deposits and traverse the endothelium to enter the bloodstream, thus, forming CTCs and pioneering the metastatic cascade. Immune cells that interact with CTCs during the initial stages of metastasis include neutrophils, natural killer (NK) cells, monocytes, macrophages, and T lymphocytes.

#### 3.1.1. Neutrophils

The recruitment of neutrophils by primary tumor is implicated in the elevated rate of intravasation in vivo [51]. In breast cancer patients, as well as in mouse models, neutrophils facilitate the intravasation of tumor cells by forming CTC-neutrophil clusters, which express higher levels of positive regulators of cell cycle and DNA replication programs than CTCs alone, leading to increased metastatic propensity and poor prognosis [22]. Activated neutrophils secrete neutrophil extracellular traps (NETs), which contain factors such as matrix-metalloprotein-9 (MMP-9), neutrophil elastase (NE), and cathepsin G (CG) that promote extracellular matrix (ECM) degradation and cellular aggregation. Evidently, these factors can promote CTC migration and invasion while enhancing proliferation and anti-apoptosis traits of CTCs at the same time [52]. Accordingly, transcriptomic analysis on peripheral blood leukocytes from treatment-naïve renal cell carcinoma patients indicated that NET formation, as indicated by elevated expression of NET formation regulators, promotes CTC viability [53,54]. Another study also demonstrated that neutrophil activation in the blood correlates with CTC survival [54]. In early-stage breast cancer patients, increased levels of NETs in the blood have been proposed as a biomarker that specifically predict the chronic risk of liver metastases [53].

#### 3.1.2. NK Cells

A study by Santos et al. demonstrated a close link between the number of CTCs and the cytotoxic activity of NK cells in the blood of breast, colorectal, and prostate cancer patients [55]. The NK cells from patients with a high number of CTCs showed diminished cytotoxicity, as compared to those isolated from patients with a low number of CTCs. This is consistent with an earlier study, where mice with low NK cell activity showed enhanced blood-borne tumor cell survival and increased incidence of metastasis [56,57].

#### 3.1.3. Monocytes and Macrophages

Monocytes and macrophages have been reported to aid in intravasation via myriad mechanisms. A three-cell complex, termed “tumor microenvironment of metastasis doorway”, composed of a perivascular macrophage, a tumor cell, and an endothelial cell, is found to act as a gateway for tumor cell hematogenous dissemination. Intravital high-resolution microscopy revealed that perivascular macrophages promote transient vascular permeability by interacting with endothelial cells, via VEGFA signaling, and consequently facilitate the intravasation of tumor cells [58,59,60]. In addition, tumor- or CTC-educated macrophages influence almost all the steps of the metastatic cascade, such as accelerating invasion, intravasation, survival in the circulation, tumor cell arrest, extravasation, as well as durable growth at distant sites [61,62]. Solid tumor-based studies suggested that CTCs intravasate into the circulatory system along with TAMs [63,64]. Additional clinical evidence of the continued interaction of macrophages with tumor cells was found in the blood circulation of cancer patients. Despite the rarity of cell types, and the shear stresses within the vascular circulation, circulating CAMLs and macrophages with pro-angiogenic capacity have been found to migrate through the circulation, attached to CTCs in 10% of late-stage patients with breast, pancreatic, or prostate cancers [11]. Together, these lines of evidence demonstrated a critical role of macrophages in metastatic intravasation.

Besides immunosurveillance, macrophages are also involved in the establishment of the premetastatic niche, particularly in the lungs. Using an intravital two-photon lung imaging system, CTCs lodged in the capillaries of the lungs were found to shed microparticles into the vasculature, driven by high shear forces within seconds of arrival. Following CTC entry, myeloid cells, such as monocytes, macrophages, neutrophils and dendritic cells (DCs), ingest these large microparticles. Of note, tumor-ingesting macrophages display an activated phenotype and pro-metastatic function, while lung-resident conventional dendritic cells showed anti-metastasis effects [65]. These findings demonstrated the complex interplay between CTCs and various immune cells.

#### 3.1.4. T Cells

There are limited investigations into the role of CD4^+^ helper T cells and CD8^+^ cytotoxic T cells in the immune surveillance of CTCs. As such, receptor activator of nuclear kappa-B ligand (RANKL) expressed by tumor-infiltrating CD4^+^ T cells are thought to stimulate RANK, expressed on CTC from breast cancer, to enhanced intravasation [66,67]. In a more recent study, CD4^+^ T cells were found to be unexpectedly involved in vasculature and immune reprogramming, thereby contributing to impeding cancer cell intravasation [68]. In that study, CD4^+^ T cell deficiency resulted in reduced immune responses, lower expression of good-prognosis angiogenesis genes (GPAGs), and higher expression of poor-prognosis angiogenesis genes (PPAGs), defined by the author. Loss of CD4^+^ T cells also altered pathways and genes that regulate vessel normalization. Two studies have illustrated the safeguarding role of CD8^+^ T cells in intravasation. Increased CTCs were found in CD8 knockout mice with breast cancer, demonstrating that loss of cytotoxic T cells promoted CTCs survival, as well as increased intravasation [68,69]. However, the mechanisms by which interaction of CTCs and T lymphocytes contribute to intravasation have not been fully clarified. Therefore, understanding the role of different T cell subsets in different stages of metastasis cascade is necessary to validate the above findings.

### 3.2. CTC Survival in Circulation

Most CTCs undergo apoptosis, owing to physical stress, through shear and tear in the circulation and anoikis [70]. Further, immune cytotoxicity eliminates the majority of CTCs in the blood [71], and deprivation of growth and survival factors outside the tumor niche also contribute to CTC death [72]. Despite these harsh survival conditions, CTCs can survive in the blood and contribute to metastasis to secondary sites by mechanisms such as cellular mutation, cytokine and growth factor stimulation, and interactions with surrounding cell types [30,73]. Here, we focus on the vast interactions between CTCs and immune cells during their voyage from the blood to dormancy and colonization in secondary sites.

The small number of CTCs that survive are able to hijack immune cells and improve their survival odds. These resistant CTCs gain enhanced metastatic seeding capability by utilizing immune cells to amplify certain traits, such as migration ability and invasiveness, as well as to modify and adapt to unfavorable circumstances. Some of the major immune cell types that interact with CTCs in circulation include neutrophils, monocytes/macrophages, lymphocytes, and NK cells [30,74].

#### 3.2.1. Neutrophils

CTC–neutrophil clusters are one of the common CTC clusters found in blood. As first responders, neutrophils are attracted to primary tumors and CTCs that produce granulocyte colony stimulating factor (G-CSF) and other neutrophil stimulating cytokines [22,75]. Initially, neutrophils induce inflammation and release hydrogen peroxide that are cytotoxic against CTCs [76]. However, as the cancer progresses, immunosuppressive TGFβ1 that originates from the primary tumor microenvironment, and platelets, can induce N2 neutrophil polarization, which aids in CTC survival and epithelial–mesenchymal transition (EMT) [77,78,79]. Additionally, CTCs can develop resistance towards neutrophil-mediated cell death, through mutation in the TLE1 gene that promotes NFkB-mediated cancer progression [80]. Neutrophils in CTC clusters are found to express adhesion factors, such as VCAM-1 and intracellular adhesion molecule 1 receptor (ICAM-1), which likely explains how neutrophils form close “piggyback” adhesions with CTCs [81,82]. This close interaction between CTCs and neutrophil enhances the activation of proliferative pathways in CTCs via crosstalk of cytokines, such as IL-6 and IL-1β, from neutrophils [75]. In addition to the pro-survival effects mentioned previously, NETs can serve as a protective cloak that bind to CTCs, via β1-integrin, to protect them from shear stress and immune cytotoxicity in the blood [83].

#### 3.2.2. Monocytes and Macrophages

Monocytes are another abundant circulating immune cell type found in CTC clusters. They patrol within the blood circulation, where they eliminate cellular debris and are a significant mediator of the inflammatory processes. Monocytes can present themselves with a diverse subset of phenotypes and functions, depending on the external stimuli in their immediate environment [84]. Shibuya et al. demonstrated that tissue repair promoting Ym1^+^ Ly6C^hi^ monocytes promoted CTC-mediated lung metastasis in the presence of systemic inflammation [85], highlighting an important role for immunosuppressive monocytes in supporting CTC metastasis. Monocytes can mature into macrophages that acquire the ability to phagocytose larger targets, including CTCs. In fact, macrophages can detect and distinguish foreign cells from self-cells through CD47, a cell surface glycoprotein that serves as a “do not eat me” signal. Furthermore, CTCs and secondary metastases are found to express high levels of CD47, suggesting that they acquire better survival advantages in blood circulation and secondary growth sites, likely by evading immunogenic responses [86].

Aside from evading phagocytosis, CTCs also take advantage of macrophages in a direct manner, through cytokine crosstalk. Tumor cells release colony stimulating factor 1 (CSF1) that recruits macrophages and polarizes them towards tissue repair-promoting M2 TAMs, which, in turn, produce growth factors like epidermal growth factor (EGF) that further stimulates tumor cells to produce CSF-1, thus, forming a positive feedback loop [87,88].

#### 3.2.3. T Cells

Effector CD4^+^ and CD8^+^ T lymphocytes exert hostile responses toward CTCs in the circulation. Further, PD-L1 expression on T cells has been found to correlate with increased CTC survival in metastatic genitourinary cancer and advanced non-small cell lung cancer (NSCLC), along with lower CD4^+^ and CD8^+^ T cell numbers [89,90]. It is suggested that PDL1^+^ CTCs are able to suppress T cell activation and, thereby, dampen the immune response against CTCs in the blood [91]. In another study, CTCs expressing elevated levels of T cell programmed cell death Fas ligand were detected in breast cancer patients, causing increased apoptosis of T cells in circulation [92]. These observations indicate that CTCs may depend on T cell deactivation and apoptosis to prolong their survival in circulation, as supported by their negative correlation with CD4^+^ and CD8^+^ T cell numbers and shorter overall patient survival [1].

Regulatory T cells (Tregs) are an immunosuppressive T lymphocyte subset that negatively regulate cytotoxic T lymphocyte activation [93]. The CTCs preferentially recruit circulating Tregs through the release of chemokines, such as CCL5, CCL22, CCL17, CXCL9 and CXCL10 [94]. Subsequently, circulating Tregs are activated by tumor-derived suppressive factors (TDSFs), including IL-10 and TGFβ1, to promote an immunosuppressive environment that limits T effector functions and contributes to CTC survival [95,96]. In addition, the RANKL, secreted by Tregs, can help promote tumor migration in RANK^+^ CTCs [66,97]. Evidently, CTC counts are positively correlated to circulating Tregs in various cancers, such as inflammatory breast cancer, hepatocellular carcinoma (HCC), and NSCLC [1,98,99].

#### 3.2.4. NK Cells

Similar to T lymphocytes, NK cells play a pivotal role in restricting CTC survival and metastasis through direct interception in circulation. The NK cells can initiate the indirect killing of CTCs through the secretion of the tumor necrosis factor-related apoptosis-inducing ligand (TRAIL) that binds to apoptotic receptors on tumor cells [100,101]. Resistant CTCs are able to mitigate this by downregulating their death receptor 5 (DR5) expression on the cell surface [102]. The secretion of granzyme and perforin from NK cells represents another form of cytotoxicity towards CTCs that is deemed more effective, capable of eliminating up to 80% of CTCs and slowing down metastatic spread [103]. Low NK cell numbers are indicated to be an independent risk factor for CTCs and their progression [1]. In recent studies, CTC clusters were shown to evade NK cytotoxicity much more effectively than single CTCs [104]. Notably, NK cell-mediated cytotoxicity is suggested to be more potent towards CTCs with partial mesenchymal-like traits, though further insight is still required to comprehend the dynamic interaction between CTCs and NK cells from the blood to metastatic colonization [105].

### 3.3. Extravasation and Colonization

As CTCs roam in the bloodstream, a small percentage of these cells will get arrested in tight capillaries, which may be a prerequisite for extravasation [106]. The CTC clusters have better odds at initiating this process due to the large size that can get embedded in tight spaces for longer periods [107]. The cancer infiltration process can be promoted through several factors, including CTC expression of surface receptors and integrins for attachment, mechanical and physical pressure, chemotactic gradient from certain secondary sites, and with aid from immune cells in clusters [108]. Here, we focus on how immune cells support and mediate the extravasation and colonization of CTCs into secondary metastasis sites.

Upon arrest, leukocytes in CTC clusters interact with endothelial cells lining the vasculature that are essential for endothelial attachment. During this process, tumor cells secrete factors such as IL-8 to promote the leukocyte expression of adhesion receptors like β2-integrin, that can bind directly to ICAM-1 and E-selectin, present on endothelial cells [109]. For instance, tumor cells that are entrapped within NETs from neutrophils act as passengers, whereby neutrophils that express CD11a (LFA1) and CD11b (Mac-1) are able to interact with ICAM-1 on endothelial cells [110]. At the same time, tumor cells produce factors that upregulate these adhesion receptors and promote migration potential in neutrophils by delaying apoptosis [111]. Monocytes and TAMs, on the other hand, secrete cytokine and chemotactic factors, such as VEGF, TGFβ1, and CCL2, to increase vessel permeability and to destroy endothelial tight junctions, thereby mediating trans-endothelial migration [112,113]. Tumor cells are also capable of following the “microtracks” generated by macrophages, as they cross the endothelial border [114].

Tumor cells that infiltrated secondary sites, hereby termed disseminated tumor cells (DTCs), have to overcome immune surveillance at the secondary site and undergo mesenchymal–epithelial transition (MET) to gain the capacity to colonize upon the local niche. In this regard, immune surveillance at different organs poses different threats, depending on the local immune composition. In breast cancer/prostate cancer-mediated bone metastases, tumor cells actively modulate the local immune niche by secreting extracellular vesicles (EVs) and factors, including VEGF, IL-6, and IL-8, that promote osteoclast differentiation and activate osteoblasts to support osteoclast activities, which causes osteolysis. Subsequently, these activated bone cells also secrete tumor growth promoting factors like TGFβ1, to advocate tumor growth and, ultimately, form a positive feedback loop [115]. These interactions enable tumor cells to undergo “osteomimicry”, where they express bone-related genes to adapt and expand upon bone sites [116]. T cells and NK cells play a prominent role in eliminating DTCs in metastatic niches [117]. In lung metastasis, tumors modify pre-metastatic niche by recruiting neutrophils to the lungs via immune–cancer crosstalk [118]. These neutrophils suppress T cell cytotoxicity via inducible nitric oxide synthase (iNOS) expression [119]. Furthermore, DTCs with altered antigen presentation characteristics are capable of minimizing T cell and NK cell cytotoxicity. In such cases, PD-L1 expression and downregulation of major histocompatibility complex I (MHC I) are modifications that allow tumors to evade NK and T cell-mediated killing, though NK cells may still recognize tumor cells with abnormally low MHC I levels [120,121]. Additionally, DTCs may recruit immunosuppressive myeloid cells to suppress NK cell activity [122]. For DTCs to fully integrate into the local niche, MET process is necessary for tumors to revert into epithelial forms. Moreover, TAMs secrete IL-35 that facilitates MET in tumor cells, through the activation of JAK2–STAT6-GATA3 signaling [123]. In the late stage of metastasis, DTCs that survive and have undergone EMT expand to form overt metastases upon acquiring sufficient growth signals in a favorable condition.

## 4. Current Detection and Isolation Strategies for CTCs

Despite steadfast advancement in CTC detection and enumeration technologies, several hurdles exist for current methodologies to accurately identify and discriminate CTCs, due to the presence of dynamic EMT phenotypes and other undiscovered CTC subtypes. Current detection strategies emphasize on discriminating CTCs, based on their biophysical characteristics and known biological markers. In the former, CTCs are physically distinguished based on their larger size and morphology, deformability, density, and electric charge [124]. Notable methods for this approach include Parsortix™, ScreenCell Cyto^®^, ApoStream^®^, CTCKey™, and Accucyte [125,126,127,128,129]. A common ground for these techniques is that they excel in having a linearized and consistent workflow. Nonetheless, apparent drawbacks include low purity and inflexibility that are highly dependent on size and morphology of CTCs. In biological marker-based detection, CTCs are recognized by immunolabeling for the surface expression of known epithelial markers, such as EpCAM and CK [130,131]. CellSearch^®^, MACS System, GEDI, GEM chip, flow cytometry, ImageStream^®^, FASTcell, and Epic are several known detection systems that utilize this approach [132,133,134,135,136,137,138,139]. Although immunolabeling enables the accurate targeting of defined CTCs, certain CTCs are revealed to have minimal to no expression of conventional epithelial markers [140]. In addition, CTCs with partial to high EMT mesenchymal traits and unorthodox phenotypes, such as the newly recognized CHCs with both epithelial and leukocyte markers, may be excluded in these conventional platforms. In consideration of this, dynamic detection strategies are required to improve CTC identification and isolation outcomes. Accordingly, newfound approaches, such as AI-based machine learning identification of CTCs, offers versatile and robust strategies for image classification. Wang et al. utilized a convolutional neural network-based machine learning algorithm to identify and detect CTCs in microscopy images [141]. Future research endeavor requires the discovery of more CTC specific traits, as well as addressing the dynamic changes and complexity of CTC identification, with respect to single cells, clusters, hybrid EMT, and other unknown subtypes.

## 5. Clinical Relevance of CTCs in Immunology

### 5.1. CTCs as a Therapeutic Target

**Figure 2:**
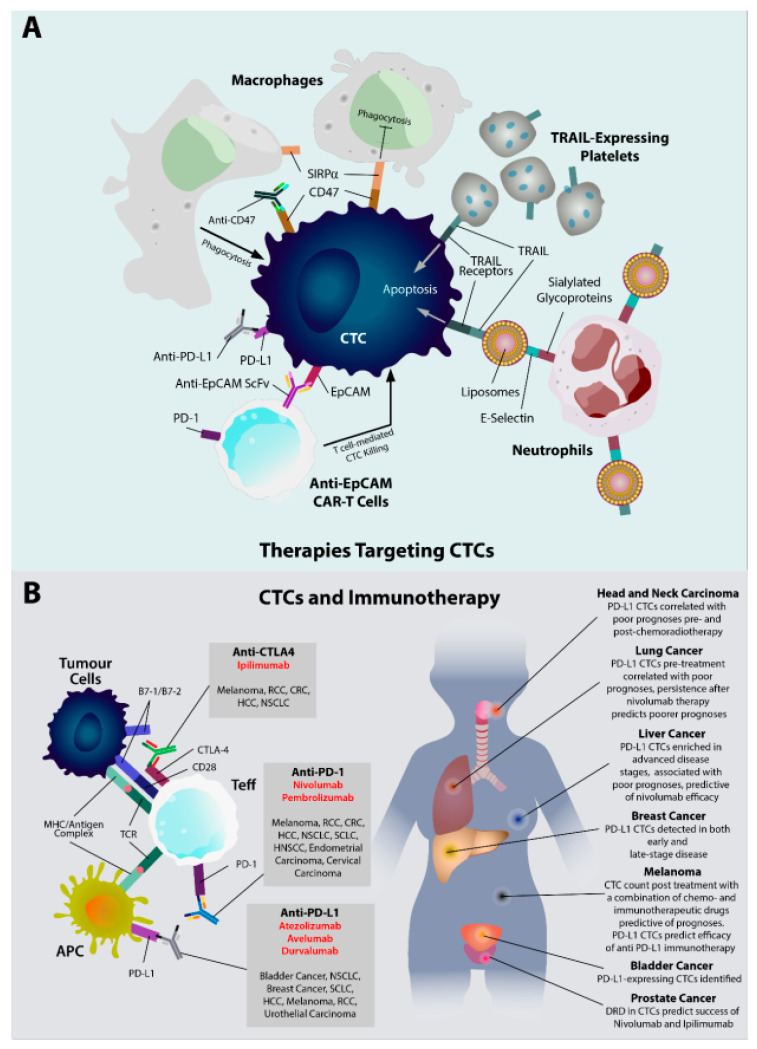
Immunotherapy and CTCs. (**A**) Therapies targeting CTCs may rely on multiple strategies, including stimulating the phagocytic elimination of CTCs by macrophages through CD47 blocking. Elimination via CAR-T cells may be targeted by homing using an anti-EpCAM ScFv. These therapies may be given in combination with anti-PD-L1 therapy. TRAIL-mediated apoptosis of CTCs may be promoted by overexpressing TRAIL on platelets or by coating circulating leukocytes with TRAIL-containing liposomes. (**B**) ICIs have been widely used as therapeutic modalities for late-stage malignancies. Three classes of ICIs are currently in clinical use for different malignancies. The role of CTCs as biomarkers for ICI therapies are increasingly studied, with the greatest focus on the relevance of PD-L1 expression for prognostics and patient stratification. Abbreviations used: CTC = circulating tumor cell, SIRPɑ = signal regulatory protein alpha, TRAIL = TNF-related apoptosis-inducing ligand, EpCAM = Epithelial cell adhesion molecule, ScFv = Single chain fragment variable, Teff = Effector T cells, CAR-T cell = Chimeric antigen receptor T cell, ICI = immune checkpoint inhibitor, RCC = renal cell carcinoma, CRC = colorectal carcinoma, HCC = hepatocellular carcinoma, NSCLC = non-small cell lung carcinoma, SCLC = small cell lung carcinoma, HNSCC = head and neck squamous cell carcinoma, TCR = T cell receptor, MHC = major histocompatibility complex, PD-1 = programmed cell death protein1, PD-L1 = programmed death ligand 1, CTLA-4 = cytotoxic T-lymphocyte associated protein-4, CD = cluster of differentiation.

Although there is substantial evidence that the immune system plays a complex role in influencing the genesis, survival and successful seeding of CTCs, surprisingly little is available on the therapeutic targeting of CTC–immune cell interactions. Although there is ample evidence that successful chemo/radio/immunotherapeutic modalities, targeting the primary tumor, also promote the elimination of CTCs [142,143,144,145], the studies outlined below are those that aim specifically at CTCs as a target of therapy, summarized in Figure 2A.

An important avenue of CTC elimination involves their phagocytosis by macrophages. However, in most solid malignancies, macrophages often present with a tumor-promoting phenotype with impaired phagocytic functions (i.e., TAMs [146]). There have been a few approaches reported to promote macrophage-dependent elimination of tumor cells, primarily via monoclonal antibody therapies. Gul et al. reported that in a murine model of melanoma, during transit through the liver, CTCs are susceptible to phagocytosis by Kupffer cells (liver macrophages) [147,148]. They reported that CTC opsonization, following monoclonal antibody therapy, using antibodies against cancer-specific antigens stimulates Kupffer cells to eliminate melanoma CTCs, via antibody-dependent phagocytosis [147]. Their report of Kupffer-dependent CTC clearance is backed by a similar report from van der Bij et al., using a murine colorectal cancer model [149]. The “do not eat me” signal, CD47, has also been targeted via monoclonal antibodies (mAbs) to reverse CTC immune evasion. Lian et al. reported that the simultaneous blockage of PD-L1 and CD47 in a murine breast cancer model reduces metastasis more effectively compared to single therapy via CTC inhibition [150,151]. Although they did not mechanistically investigate the cause of CTC reduction, other groups have shown that CD47 blockage promotes macrophage-dependent phagocytosis [152,153].

Another approach at CTC elimination utilizes the close association between CTCs and platelets during circulatory transit. Li et al. engineered platelets that overexpress membrane-bound TRAIL to promote CTC apoptosis [154]. They demonstrated that in murine models for breast and prostate cancers, recruitment of TRAIL-overexpressing platelet to CTC clusters (as part of the CTC “cloaking” strategy) resulted in reduced metastasis and CTC viability [154]. A more recent study by Ortiz-Otero et al., using TRAIL-overexpressing platelets, also reported similar results with primary-tumor-derived CTCs [155]. Another reported approach using TRAIL, involves generating TRAIL and E-selectin-containing liposomes [156]. These liposomes bind circulating leukocytes via their E-selectin (effectively coating these leukocytes with the accompanying TRAIL molecules with monocytes, neutrophils and NK-cells, showing the highest level of coating). These cells are then primed to promote TRAIL-mediated CTC apoptosis and were shown to successfully reduce lung metastasis in a murine colorectal cancer model [156]. Recent research has also suggested that EpCAM can be utilized as a target antigen for CAR-T cell therapy, potentially selectively eliminating CTCs. However, their reported efficacy seems to also be accompanied by an unfavorable toxicity profile [157].

Immunotherapeutic modalities are often prescribed as second or subsequent-line drugs, usually prescribed for late-stage disease, when CTCs are usually also at their peak abundance [158]. Overall, larger studies are required to derive stronger conclusions on the utility of CTCs to predict ICI efficacy and to refine patient stratification. For a more in-depth review of CTC in immunotherapeutics, Leone et al. and Schuster et al. also summarized the clinical evidence [2,6]. A list of more recent clinical trials can be viewed in Table 1, updated to November 2021.

### 5.2. CTCs as Diagnostic and Therapeutic Biomarkers

**Figure 3:**
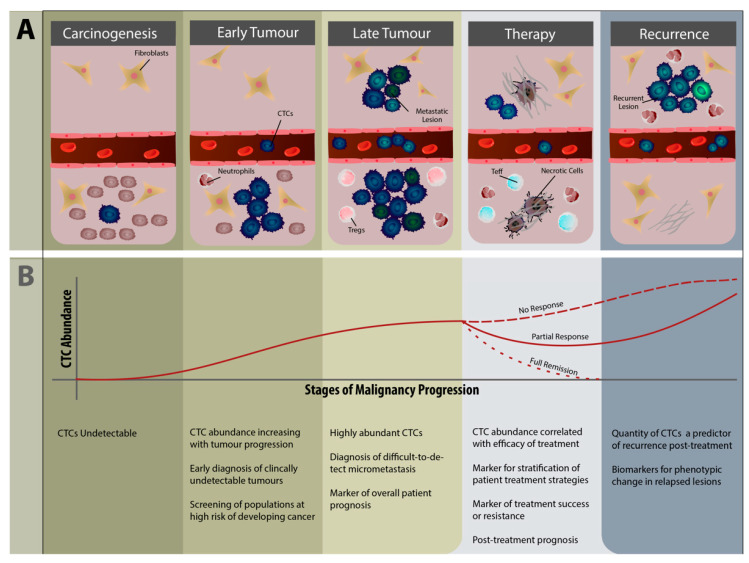
The Role of Circulating Tumor Cells in the Clinical Setting. (**A**) CTCs are released during the progression of tumor growth, being detectable even during the early stages of malignancies. At later stages, CTCs show higher heterogeneity and may reflect the mutational profiles of the primary tumor or metastatic lesions. The heterogeneity of CTCs may also aid with the survival of smaller resistant populations during treatment, which then promotes disease recurrence. (**B**) Once CTCs are released into the circulation during the early stages of malignancies, they may be useful as early diagnostic markers. As abundance increases during later disease stages, more detailed phenotypic assessments may inform of potential tropism to certain organs for metastasis as well as long-term patient prognoses. Prior to and during treatment, stratification strategies, treatment efficacy and post-treatment prognosis may be informed via CTC analysis with CTC abundance typically being inversely correlated with treatment success. Finally, likelihood of tumor recurrence may also be predicted via CTC analysis. Abbreviations used: CTC = circulating tumor cell, Treg = regulatory T cells, Teff = Effector T cells.

In contrast to the scarcity of evidence found for CTC-targeted therapies, a much more well-researched area of CTC biology concerns their utility as biomarkers (a representative surrogate for primary and metastatic tumors, used as clinical guides for patient stratification, prognostic determination, or therapeutic evaluation [159]). Often grouped together with circulating tumor DNA (ctDNA), as cancer-specific biomarkers obtained from the blood, liquid biopsies are much easier and less invasive to obtain compared to tumor biopsies [160]. The value of CTC liquid biopsies in cancer prognostics has been well documented for many solid tumors in the clinical setting, summarized in Figure 3. The EpCAM^+^ CTC-based CellSearch test is currently the only FDA-approved clinical platform for use in prognostics and patient stratification in metastatic breast, prostate and colorectal cancer [131]; however, this have also have been used to research other malignancies [161].

Although primarily associated with metastasis and late-stage malignancies, CTCs have also been demonstrated to be present in the early stages of many cancers [162], as even stage I epithelial tumors have been shown to release CTCs [163,164]. This has driven research into the utility of CTCs as a highly specific early diagnostic tool. In patients with risk factors to the development of malignancies, CTCs may be released in small quantities by tumors which are not macroscopically detectable [165]. This approach allows for these clinically asymptomatic tumors to be detected early. Its role in screening has been demonstrated for early detection of lung cancer in patients with COPD [166], as well as for early or asymptomatic HCC [167], prostate cancer and breast cancer [168].

With the use of scRNA-seq becoming increasingly common, deeper investigations into the transcriptomic diversity of CTCs have been some of the more recent highlights in the field. Using metastatic HCC samples, D’avola et al. used scRNA-seq to identify distinct expression profiles in different HCC CTC populations, showing that some CTCs show upregulation of angiogenesis-related genes, and in others, KRAS and G2M checkpoint genes [169]. In metastatic breast cancer, De Luca et al. showed that not only do CTCs exhibit significant interpatient heterogeneity, single CTCs may possess unique mutations which will be lost in bulk analysis [170]. In the same paper, they also reported that the mutational landscape of CTCs may be completely altered following treatment [170]. A recent publication by Sun et al. described a high degree of spatial heterogeneity of the CTC transcriptional landscape in HCC via scRNA-seq [96]. They identified a large number of transcriptomic differences between CTCs drawn from liver efferent (representing primary tumor heterogeneity) and efferent (representing CTC adaptation in circulation) vessels. They also identified CCL5 as being increasingly upregulated, the longer CTCs circulate as an immune evasion mechanism, by recruiting Tregs in circulation [96]. Single-cell analysis also allows for better discrimination of CTC subpopulations, classifying them based on their transcriptional signatures or drug resistance. For example, in metastatic pancreatic cancer, Ting et al. was able to segregate CTCs into three transcriptionally distinct groups (classical CTCs, platelet-adherent CTCs and a subset, exhibiting high proliferation [171]). Aberrant signaling pathways in small populations of CTCs may also contribute to partial drug resistance. Miyamoto et al. reported that increased noncanonical Wnt signaling, in a population of pancreatic cancer CTCs, allowed their survival against antiandrogenic agents, which potentially allows for relapse or treatment failure [172].

A more recent development in the field is research into the predictive and evaluative value of CTCs in immunotherapeutic regimens (Figure 2B). The CTCs hold substantial promise as immunotherapeutic biomarkers due to their progressive enrichment during disease progression. Immunotherapeutic modalities are often administered as second or subsequent-line drugs, usually prescribed for late-stage disease, when CTCs are at their peak abundance [158]. As mentioned previously, PD-L1 has been discovered on CTCs, and this has driven interest in their role during immune checkpoint inhibitor (ICI) therapy. Although most of the research in the field have been focused on ICI therapies, some recent work on their utility in adoptive cell transfer and DC immunotherapy will also be highlighted.

Immune checkpoint inhibitor therapy has been the most successful immunotherapeutic modality to date, with anti-CTLA-4 and anti-PD-1/PD-L1 mAbs already in clinical use [173]. Additionally, CTC quantification and qualitative analysis in patients receiving ICIs suggests that their analysis pre- and post-treatment may be prognostically useful in predicting overall survival and treatment response. In malignant melanoma, two separate groups used a combination of chemotherapeutic and ICI agents to investigate the prognostic utility of CTCs and concluded that changes to CTC counts post-treatment is a good marker of prognosis [174,175]. More detailed qualitative analysis of melanoma CTCs showed that the presence of PD-L1^+^ CTCs pre-treatment predicts sensitivity to anti-PD-1 ICIs [176,177]. Conversely, expression of Catenin Beta 1 on CTCs is a predictor of resistance to ICI therapy [178]. More in-depth analysis of melanoma CTCs using RNA signatures have also been recently reported [179].

In lung cancer, the presence of PD-L1 on NSCLC CTCs was reported as a predictor of lower overall survival (OS) [180], although a different group did not find a statistically significant result [181]. Another group reported poorer prognoses of patients with PD-L1^+^ CTCs and that persistence of PD-L1^+^ CTCs after nivolumab therapy is associated with worse overall survival [182]. Further studies by Guibert et al. and Dhar et al. showed that higher PD-L1^+^ CTC count pre-treatment is associated with worse progression-free survival (PFS) and that CTCs express higher levels of PD-L1 compared to primary tumors [183,184]. Although ICIs have not been extensively studied for breast carcinomas, Mazel et al. demonstrated the presence of PD-L1 on 68% of breast cancer CTCs [185]. Expanding on this, Schott et al. found that PD-L1 positive CTCs are present on both earlier and metastatic disease stages [186] and reported that one patient showed a reduction in PD-L1^+^ CTCs, with successful nivolumab and ipilimumab treatment.

Other malignancies with CTCs being studied as biomarkers for immunotherapy include prostate, bladder [187], head and neck squamous cell carcinoma (HNSCC) and HCC. In metastatic prostate cancer, with CTCs expressing AR-V7, Boudadi et al. reported that nivolumab and ipilimumab are efficacious only when tumors also show DNA-repair deficiencies [188]. In HNSCC, the presence of PD-L1^+^ CTCs in HNSCC pre-treatment was associated with poorer prognoses [181] and lower PFS and OS post-chemoradiotherapy [189]. In HCC, the presence of PD-L1^+^ CTCs is a predictor of worse OS but predicts a positive response to nivolumab treatment [190].

Studies addressing the role of CTCs in DC vaccination and ACT therapies are much less prevalent than for ICIs, with only the DC vaccine Sipuleucel-T in clinical use for prostate cancer [191]. Rekoske et al. found that PD-L1 expression on CTCs increased after administration of Sipuleucel-T and is associated with sustained T-cell responses and longer PFS [192]. In autogeneic NK-cell adoptive cell transfer for breast cancer and NSCLC, CTC quantity is negatively correlated with therapeutic efficacy [193,194].

Although momentum has been growing in efforts to bring relevance to CTCs, as both targets and biomarkers of cancer therapy, much work remains. Most studies targeting CTCs via immunotherapies are still at the preclinical stage and clinical biomarker studies often involve small study populations. Deeper investigations are still required to fully translate the mechanistic findings of CTC immunology onto the bedside.

### 5.3. Future Avenues of CTC Research

Efforts to understand CTC complexity have recently been supported by the increasing accessibility of single-cell transcriptomic analysis. In addition to the high spatiotemporal heterogeneity seen in primary lesions, CTCs may undergo various degrees of EMT, resulting in an even more varied transcriptome [10]. Further, CTC clusters have been shown to be composed of CTCs at different stages of EMT, where individual CTCs possess specialized roles in causing successful metastasis [10,195]. Appreciating the diversity of CTCs may illuminate more complex immune–CTC interactions, although research on the topic is very limited at the time of writing. Notably, Sun et al. demonstrated the spatial heterogeneity of CTCs by investigating transcriptomic differences between HCC CTCs drawn from liver efferent and afferent vessels (reflecting newly-released and circulation-adapted CTCs, respectively [96]). They identified CCL5 production as a CTC immune evasion strategy by recruiting Tregs in circulation, with levels of CCL5 increasing, the longer CTCs are in circulation [96]. Single-cell analyses also allow for better discrimination of CTC subpopulations, classifying them based on their transcriptional signatures [171] or drug resistance [172]. Identifying the immunological significance of these subpopulations may be an interesting avenue of research.

With the recent advent of personalized neoantigen cancer vaccines [196], CTCs may find utility for neoantigen screening. Neoantigen vaccines have been shown to be successful modalities for multiple cancer types, with very favorable toxicity profiles, due to their high specificity [197,198,199]. Although these studies relied on the sequencing of primary tumor biopsies, performing neoantigen screening via liquid biopsies may become increasingly feasible. A recent study in NSCLC, by Jia et al., showed that neoantigens identified from primary tumor biopsies were also detectable on ctDNAs. They then tracked the success of ICI therapy via fluctuations in neoantigen ctDNA abundance [200]. This suggests that neoantigen sequences can be tracked from liquid biopsies. However, as of the time of writing, there are still no reports on neoantigen detection using CTCs. A large caveat to this approach, however, is that there may be varying levels of similarity between the transcriptomic profile seen in CTCs and primary or metastatic lesions [10,201,202]. As such, extrapolating any neoantigen findings in CTCs to primary or metastatic lesions requires much scrutiny to establish their clinical significance.

Another field that is rapidly developing is the use of machine learning methods for CTC analysis, which provides a fully automated and robust platform for CTC enumeration. These methods can identify and characterize CTCs in heterogeneous liquid biopsies in a reproducible and accurate manner [203,204]. The CTC counts were obtained by combining autoencoding convolutional neural networks (CNN) with advanced visualization techniques to predict overall survival in metastatic breast cancer patients [205]. The CNN have also been employed to detect and classify rare CTC cells from metastatic renal cell carcinoma patients [141]. Likewise, deep learning radiomics was employed for CTC counts to predict disease recurrence in early-stage non-small cell lung cancer patients, treated with stereotactic body radiation therapy [206].

## 6. Conclusions

Multiple studies have established the important role of CTCs during the metastasis process. CTCs interact with major immune cells, including neutrophils, monocytes, platelets, T cells and NK cells, to survive in circulation, evade destruction, proliferate, and finally colonize secondary sites. The CTCs interact with immune cells via cytokines and chemotactic factors. Although single CTCs are easily destroyed in circulation, cluster formation of CTCs with immune cells has allowed for more resistant CTCs, with enhanced migratory and invasive abilities that promote metastatic seeding. Various studies have provided valuable insights into the role of CTCs during metastasis. However, a number of questions still remain unanswered. For example, the dynamic interaction between CTCs and NK cells from circulation to metastatic colonization and the role of different T cell subsets in different stages of metastasis cascade. Key future research directions include understanding CTC heterogeneity, the use of immunotherapy to target CTCs, and the utility of CTCs in neoantigen screening. A major hurdle in CTC research is the rarity and fragility of these cells in liquid biopsies. With the advent of scRNA sequencing techniques, allowing for analysis at single cell level, these limitations can be overcome.

## Figures and Tables

**Table 1:** Current clinical trials for CTCs in patients undergoing cancer immunotherapies. The list is based on the ClinicalTrials.gov database as of November 2021.

Trial Number	Study Title	Enrollment No.	Disease	Interventions	Status	Start/Completion Date (m/d/y)	Country
NCT03986463	CIrculating Tumor DNA in Lung Cancer (CITaDeL): Optimizing Sensitivity and Clinical Utility	40	Lung Neoplasms NSCLC	ctDNA	Completed	5/1/2019 - 12/31/2020	London Regional Cancer Program
NCT02827344	Feasibility Study of PD-L1 Expression Analysis on Circulating Tumor Cells by Immunocytochemistry and MDSCs Level Evolution Analysis in NSCLC Treated With PD- L1 or PD1 Inhibitor	200	Lung Cancer	Blood sample collection for CTCs and MDSCs analysis	Recruiting	10/1/2015	Larrey Hospital Toulouse, France
NCT03481101	WHENII—Early Response Evaluation With FDG- PET/CT and Liquid Biopsy in Patients with NSCLC	60	NSCLC	PET/CT CTCs ct DNA	Recruiting	2/28/2018	University Copenhagen
NCT03926260	Early Assessment of Response to Treatment of Metastatic Lung Tumors Based on Circulating Tumor DNA	100	Metastatic NSCLC	ctDNA analysis	Recruiting	6/27/2019	Marie MARCQ La Roche-sur-Yon, France
NCT04791215	Circulating Tumor DNA Alterations in Non- small Cell Lung Cancer Patients Treated with Pembrolizumab	37	NSCLC	Observational	Recruiting	2/1/2020	Columbia University, United States
NCT05091190	Immunotherapy Clearance and Phenotype of Circulating Tumor Cells in Lung and Head and Neck Cancers	60	Metastatic NSCLC Metastatic Head and Neck Cancer	Blood draws	Not yet recruiting	October 2021	Croix Rousse Hospital, France
NCT04053725	A Prospective Study of Blood Circulating Tumor DNA for the Prediction of Efficacy in Immunotherapy for Advanced Gastric Cancer.	200	Stomach Neoplasms	NP	Unknown	2/1/2019	Cancer center of Sun Yat-sen University, China
NCT04944173	A Study of Durvalumab and Stereotactic Radiotherapy for Stage I Non-Small Cell Lung Cancer (SCION)	94	NSCLC Lung Cancer, Stage I Lung Adenocarcinoma, Stage I Lung Squamous Cell Carcinoma Stage I	Durvalumab Stereotactic Body Radiotherapy ct DNA assay	Phase 2 Not yet recruiting	June 2021	BC Cancer—Kelowna, Canada
NCT04966663	From Liquid Biopsy to Cure: Using ctDNA Detection of Minimal Residual Disease to Identify Patients for Curative Therapy After Lung Cancer Resection	66	NSCLC	Nivolumab Pemetrexed Gemcitabine Cisplatin Carboplatin ctDNA blood test	Phase 2 Not yet recruiting	11/1/2021	Princess Margaret Cancer Centre, Canada
NCT04993014	Circulating Tumor Cells and Treatment De-escalation After Neoadjuvant Therapy for HER2 Positive Breast Cancer	80	Breast Neoplasms HER2+ Breast Cancer	Pertuzumab Trastuzumab CTCs	Phase 2 Recruiting	3/1/2021	A.C. Camargo Cancer Center, Brazil
NCT04168931	Efficacy of Adding Trastuzumab to Standard Chemotherapy in Patients with Advanced HER2-negative Gastric Cancer and HER2 Positive Expression in Circulating Tumor Cells	85	Gastric Cancer Stage IV	Trastuzumab	Phase 2 Recruiting	1/1/2020	AC Camargo Cancer Center, Brazil
NCT04367311	Adjuvant Treatment with Cisplatin-based Chemotherapy Plus Concomitant Atezolizumab in Patients with Stage I (Tumors ≥ 4 cm), IIA, IIB, and Select IIIA [T3N1-2, T4N0-2] Resected NSCLC and the Clearance of ctDNA	100	NSCLC	Atezolizumab Docetaxel Cisplatin Pemetrexed	Phase 2 Recruiting	5/22/2020	Northwestern University, United States

Abbreviations: cell-free circulating tumor deoxyribonecleic acid (ctDNA); circulating tumor cells (CTCs); myeloid-derived suppressor cells (MDSCs); not provided (NP); non-small cell lung cancer (NSCLC).

## Data Availability

Not applicable.
